# Disparities in COVID-19 Mortality in High-Minority Nursing Homes

**DOI:** 10.1093/geroni/igab046.2012

**Published:** 2021-12-17

**Authors:** Justin Lord, Robert Weech-Maldonado, Ganisher Davlyatov, Akbar Ghiasi, Gregory Orewa

**Affiliations:** 1 Louisiana State University at Shreveport, Bossier City, Louisiana, United States; 2 University of Alabama at Birmingham, Birmingham, Alabama, United States; 3 Birmingham, Alabama, United States; 4 University of the Incarnate Word, San Antonio, Texas, United States

## Abstract

Racial/ethnic disparities in healthcare have been highlighted by the recent COVID-19 pandemic. Minorities continue to utilize nursing home services at a higher rate than White residents, contributing to existing health inequity concerns. This study examined the relationship between nursing home racial/ethnic mix and COVID-19 resident mortality using the CMS Nursing Home COVID-19 Public File. As of October 25, 2020, high minority nursing homes reported 6.5 COVID-19 deaths as compared to 2.6 deaths for nursing homes that had no racial/ethnic minorities. Four nested sequential negative binomial regressions were used to model the relationship between racial/ethnic disparities in COVID-19 deaths and the separate contributions of facility-level resident characteristics (percent of females, percent of residents 65 years and older, percent of residents with congestive heart failure, hypertension, and obesity, and the average level of residents’ acuity), resource availability (nursing homes’ payer-mix, occupancy rate, county-level Social Deprivation Index, and nursing home location), and other organizational characteristics (nursing home for-profit status, chain affiliation, and self-reported nursing, clinical, aides, and other staff shortages). After controlling for interstate differences, facility-level resident characteristics, resource availability, and organizational characteristics, high-minority nursing homes had 61% more COVID-19 deaths (Incidence Rate Ratio [IRR] = 1.61; p < 0.001) as compared to nursing facilities with no minorities. From a policy perspective, nursing homes, that serve primarily minority populations, may need additional resources, such as, funding for staffing and equipment in the face of the pandemic. The COVID-19 pandemic has sharpened the focus on healthcare disparities and societal inequalities in the long-term care.

